# Avalanching strain dynamics during the hydriding phase transformation in individual palladium nanoparticles

**DOI:** 10.1038/ncomms10092

**Published:** 2015-12-11

**Authors:** A. Ulvestad, M. J. Welland, S. S. E. Collins, R. Harder, E. Maxey, J. Wingert, A. Singer, S. Hy, P. Mulvaney, P. Zapol, O. G. Shpyrko

**Affiliations:** 1 Department of Physics, University of California-San Diego, La Jolla, California 92093-0319, USA; 2 Materials Science Division, Argonne National Laboratory, Argonne, Illinois 60439, USA; 3 School of Chemistry & Bio21 Institute, University of Melbourne, Parkville, Victoria 3010, Australia; 4 Advanced Photon Source, Argonne National Laboratory, Argonne, Illinois 60439, USA; 5 Department of Nano Engineering, University of California-San Diego, La Jolla, California 92093-0319, USA

## Abstract

Phase transitions in reactive environments are crucially important in energy and information storage, catalysis and sensors. Nanostructuring active particles can yield faster charging/discharging kinetics, increased lifespan and record catalytic activities. However, establishing the causal link between structure and function is challenging for nanoparticles, as ensemble measurements convolve intrinsic single-particle properties with sample diversity. Here we study the hydriding phase transformation in individual palladium nanocubes *in situ* using coherent X-ray diffractive imaging. The phase transformation dynamics, which involve the nucleation and propagation of a hydrogen-rich region, are dependent on absolute time (aging) and involve intermittent dynamics (avalanching). A hydrogen-rich surface layer dominates the crystal strain in the hydrogen-poor phase, while strain inversion occurs at the cube corners in the hydrogen-rich phase. A three-dimensional phase-field model is used to interpret the experimental results. Our experimental and theoretical approach provides a general framework for designing and optimizing phase transformations for single nanocrystals in reactive environments.

## Abstract (web summary)

Phase transformations in nanoparticles can have a large effect on the performance of electrochemical devices and are strongly determined by parameters such as surface energy and faceting. Here, the authors study the hydriding phase transformation in individual palladium nanocubes and uncover individual structure-function relationships.

## Graphical abstract

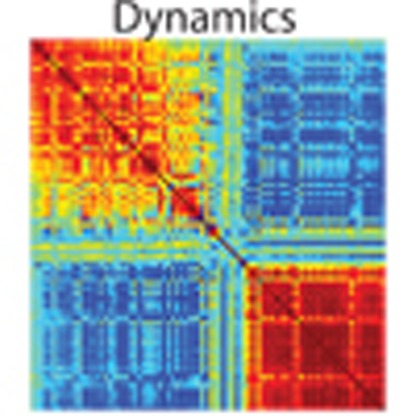

The palladium hydride system is a prototypical model useful for studying the fundamentals of solute intercalation, interaction and phase transformations, relevant to a broad class of systems^1^,^2^,^3^. The system is also technologically important in many energy-based applications, including hydrogen purification and storage, memory switching and hydrogen embrittlement^4^,^5^. Consequently, the system has been intensely studied^6^. Palladium initially forms a dilute interstitial solid solution with H, known as the α phase, whose lattice constant expands slightly as the H concentration increases. As the H concentration increases further, a phase transformation to the lattice-expanded β phase occurs^1^. The β phase lattice then expands further as more hydrogen is incorporated^5^. The Pd sublattice maintains the face-centered cubic structure in both phases. Despite intense investigation^7^,^8^,^9^, there are many conflicting results and open questions surrounding both the new phase nucleation (that is, coherent versus incoherent α–β interface) and growth (that is, sharp transition or two-phase coexistence) in addition to the role of surface effects. Ensemble measurements suggest that the phase transformation from the α to β phase is continuous^10^ and exhibits two-phase coexistence^11^,^12^, consistent with the observed isotherm plateau and the Gibbs phase rule^13^. However, a recent single-particle study discovered sharp α–β transitions in 20 nm nanocubes^14^. Although recent single-particle measurements eliminated size diversity^14^,^15^, strain information, which is crucially important in understanding the catalytic and solubility properties of Pd nanocrystals^16^,^17^,^18^,^19^, was unresolved. An understanding of three-dimensional (3D) strain fields during the hydriding phase transformation, resolved via coherent X-ray diffractive imaging (CXDI), could thus aid in developing improved catalysts, storage media and sensors in the future.

CXDI is an X-ray imaging technique capable of resolving 3D strain distributions in reactive environments under both *in situ* and operando conditions^20^,^21^,^22^,^23^. In Bragg geometry, scattered coherent X-rays are recorded in the far-field using X-ray sensitive area detectors. Phase-retrieval algorithms^24^ are then used to reconstruct the 3D electron density and lattice displacement fields in single nanocrystals^23^,^25^,^26^,^27^. The penetrating power of high-energy X-rays makes CXDI an ideal probe for studying operating devices^28^, while the ability to resolve the full 3D displacement field is essential for understanding the complex role of crystallographic facets^29^, defects^30^,^31^ and surface effects^29^ in nanoscale dynamics.

In this article, we use CXDI to reveal strain evolution during the hydriding phase transformation in individual palladium nanocubes. By comparing experimental results with a 3D phase-field model of the process, we corroborate strain distributions with concentration distributions. The hydrogen-poor phase strain is dominated by a residual hydrogen-rich surface layer while the hydrogen-rich phase strain is dominated by elastic effects. Finally, the time–time displacement field correlations exhibit signs of aging and avalanching. We begin with the discussion of the hydrogen-rich β phase followed by the discussion of the hydrogen-poor α phase (which has undergone one cycle of hydrogen exposure) before concluding with the phase transformation dynamics.

## Results

### Experimental description

**Figure 1:**
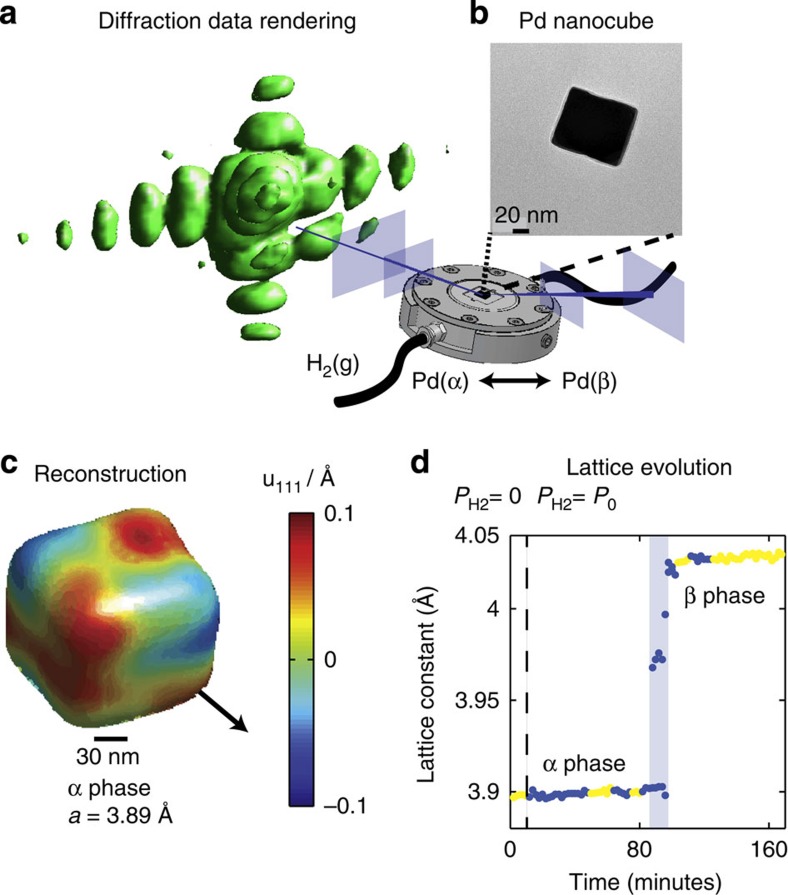
Imaging the hydriding phase transformation in Pd nanocubes with coherent X-rays. (**a**) Isosurface rendering of the diffraction data obtained from an α phase Pd nanocube. (**b**) Scanning electron microscopy image of a Pd nanocube on a silicon substrate. (**c**) Reconstructed [111] displacement field projected onto an isosurface drawn at constant electron density. The [111] direction is shown by a black arrow. (**d**) The single-particle lattice constant as a function of time, with the dashed black line indicating when the partial pressure of H_2_(g) is increased from 0 to *p*_0_. The shaded region indicates that two diffraction peaks were observed on the detector. The yellow highlighted points correspond to the reconstructions discussed in Figs 2, 3, 4.

The experimental set-up is shown schematically in Fig. 1. Focused coherent X-rays are incident on a gas environmental X-ray cell (Supplementary Fig. 1) that contains palladium nanocubes^32^ on a silicon substrate (Fig. 1b and Supplementary Fig. 2). Figure 1a shows an isosurface rendering of a (111) diffraction pattern from an individual palladium nanocube. The diffraction intensity is proportional to the Fourier transform of the electron density^33^,^34^ and thus is similar to the Fourier transform of a cube. Well-defined fringes indicate adequate oversampling of the diffraction intensity. Two-dimensional slices of the raw experimental data for both an α and β phase nanocube are shown in Supplementary Fig. 3.

From the 3D coherent diffraction data, we reconstruct both the 3D distribution of Bragg electron density, *ρ*(**r**), and the 3D lattice displacement field along [111], *u*_111_(**r**) with 16 nm resolution defined by the phase-retrieval transfer function (Supplementary Fig. 4). In Bragg geometry, the reconstructed electron density is due to atomic planes that satisfy the Bragg condition and is called the Bragg electron density. Thus, portions of the crystal that do not satisfy the Bragg condition appear missing (such as twin domains^35^), although the physical electron density is present. Figure 1c shows a representative example of both the shape and *u*_111_ displacement field for α phase nanocubes. We observe no evidence of dislocations, which manifest themselves as singularities in the displacement field^30^,^31^.

**Figure 2:**
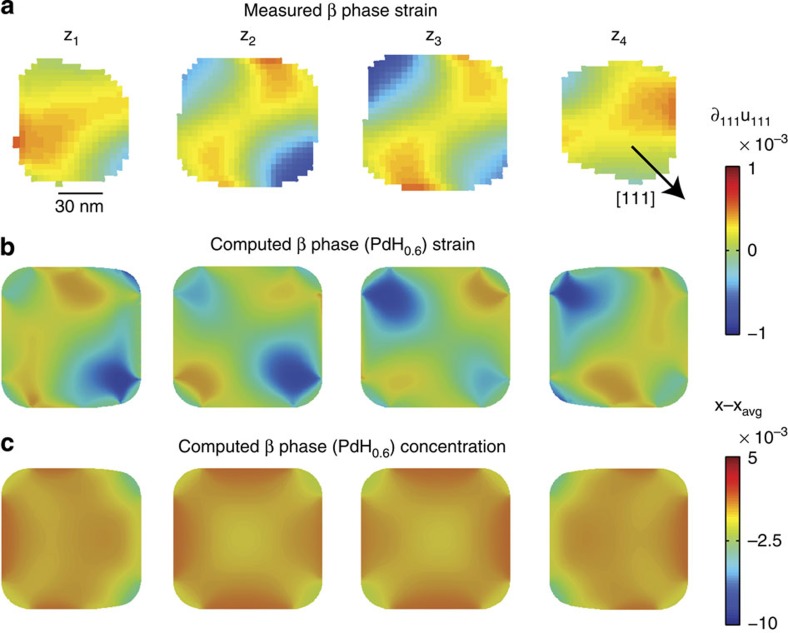
Strain field cross-sections in the β phase compared with phase-field calculations. (**a**) The measured compressive/tensile strain distribution, ∂_111_*u*_111_, at four cross-sections taken at spatial locations throughout the cube as indicated in Supplementary Fig. 6. The black vector shows the projection of the [111] vector in the chosen slice. (**b**) The expected ∂_111_*u*_111_ computed by the phase-field model. (**c**) The corresponding compositional inhomogeneity within the nanocube.

The black arrow indicates the [111] direction is through a cube corner, which is consistent with {100} cube faces. The displacement field before and after 4 h of X-ray exposure shows negligible changes (Supplementary Fig. 5). Figure 1d shows the evolution of the single-particle's average lattice constant, determined from the scattering angle of the maximum intensity location in the 3D diffraction data, as a function of time. At 10 min, a flow of H_2_(g) is initiated that causes the particle to transform fully to the β phase ∼110 min later. We further investigated the displacement field structure by calculating a component of the strain field, the compressive/tensile component along [111], in both the α and β phase. Figure 2 shows the β phase strain distributions for a single nanocube obtained by averaging over the yellow highlighted states in Fig. 1d from 128 to 166 min.

Palladium nanocube strain is expected to be due to elastic effects arising from surface stress and, potentially, compositional inhomogeneity. Elastic strain is induced by the cube attempting to minimize its surface energy by contracting its corners inward, evolving towards a spherical geometry^36^. As Fig. 2 shows, the corners along the projection direction, in this case [111], will be compressed. Interstitial hydrogen also induces strain by expanding the palladium lattice constant, which can be modelled through Vegard's law^37^.

### Construction of the phase-field model

To understand the contributions of these two effects, we constructed a phase-field model based on the original Cahn–Hilliard work^38^,^39^,^40^. The free energy of the particle is described by the free-energy functional:


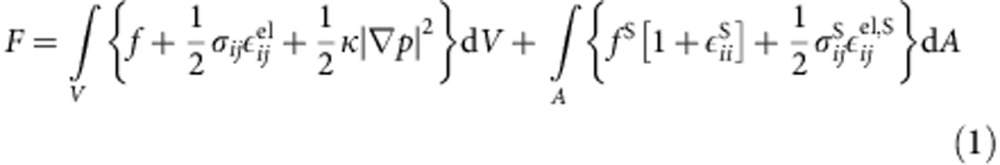


where *f* is the free-energy density containing both the enthalpy and the entropy, 
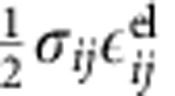
 is the elastic energy density, *p* is the local fraction of the β phase and 
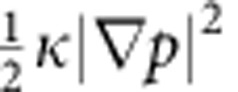
 is the gradient energy density^41^. The surface contribution includes the surface energy (in the Lagrangian system), which is the product of an undilated surface energy and a surface dilation term, and also includes an additional stress accounting for the change in surface energy with strain. Phase-field models have recently found use in describing the properties of nanoscale insertion materials for Li-ion batteries^42^,^43^. In particular, dynamics in phase-separating cathodes, such as LiFePO_4_ and LiNi_0.5_Mn_1.5_O_4_, during charge and discharge can be well described by appropriately accounting for a variety of effects, including surface wetting^44^, elastic energy^45^ and chemical kinetics^46^. For more details, please refer to the Phase-Field Model section in the Methods section.

### Strain in the hydrogen-rich phase

Figure 2a shows the strain distributions measured in a β phase nanocube at four cross-sections at the spatial positions in the cube indicated in Supplementary Fig. 6. The strain map shown is computed from an average of the reconstructions corresponding to the highlighted points from 128 to 166 min in Fig. 1d. The β phase strain distribution agrees well with the strain distribution computed by the phase-field model shown in Fig. 2b. The primary differences occur in slices z_1_ and z_4_, which are near the cube boundary along the beam direction. This could indicate deviations of the particle geometry from an ideal cube shape near these surfaces. Figure 2c shows the corresponding relative hydrogen concentration map. Although a uniform hydrogen concentration field was chosen as the initial condition for the simulation, the minimum energy configuration is an inhomogeneous concentration field. The compositional inhomogeneity manifests itself at the cube corners, which have relatively less hydrogen compared to the cube faces. The average composition for the particle in the simulation is PdH_0.6_.

The other β phase particles that were measured exhibited similar strain distributions (for an additional particle, see Supplementary Fig. 7) and thus the β phase is primarily dominated by elastic effects with small compositional inhomogeneity. We now discuss the strain distribution observed in the α phase.

### Strain in the hydrogen-poor phase

**Figure 3:**
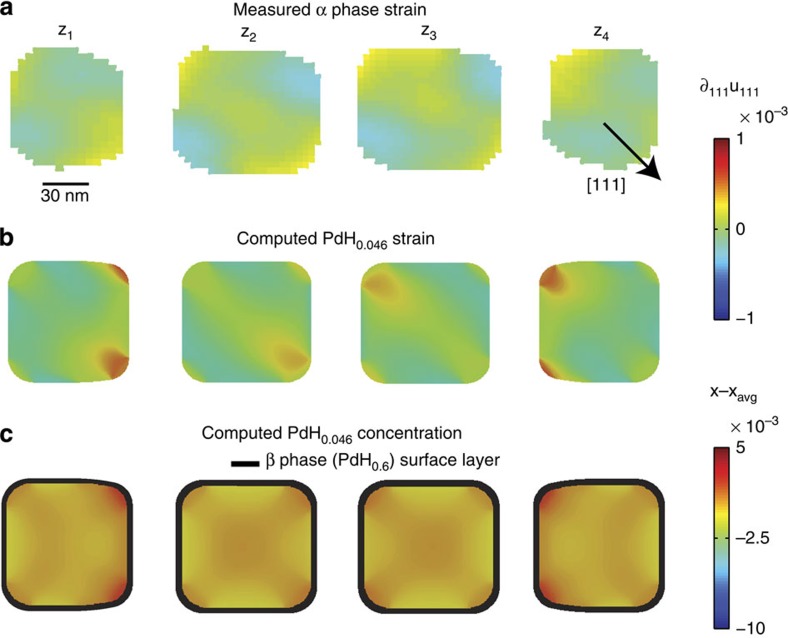
Strain field cross-sections in the α phase compared with phase-field calculations. (**a**) The measured compressive/tensile strain distribution, ∂_111_*u*_111_, at four cross-sections. The cross-sections are taken at locations indicated in Supplementary Fig. 6. The black vector shows the [111] projection in the chosen slice. (**b**) The ∂_111_*u*_111_ strain distribution computed by the phase-field model. (**c**) The corresponding compositional inhomogeneity within the nanocube and the hydrogen-rich surface layer.

Figure 3a shows the strain distribution measured in the α phase for the same nanocube as in Fig. 2 at the same four cross-sections (Supplementary Fig. 6). The strain map shown is computed from an average of the reconstructions corresponding to the highlighted points from 2 to 10 min in Fig. 1d. This particle was previously exposed to hydrogen and dehydrided at room temperature for 3 h before the measurements were made. The strain distribution is inverted (opposite in sign) from the elastically dominated distribution shown in Fig. 2b, implying the corners are stretching outwards along the body diagonals, forming a star-like shape. This shape evolution increases the surface area of the particle in contrast to the behaviour in the β phase. We used the phase-field model to investigate strain and hydrogen distributions at low average hydrogen concentrations in the presence of a hydrogen-rich surface layer.

Figure 3b,c shows the phase-field results most consistent with the observed strain distribution. We explored two possible models (surface wetting and surface monolayer) for the observed α phase strains. In both models, the qualitative effect is to cause expansion along the body diagonal corners defined by the scattering vector, as shown in Fig. 3a,b. Given the additional parameters required by the surface wetting effect (including assumptions about the surface energy dependence on concentration and the interface width), we chose to present results from the simpler model, which is similar to the model discussed in ref. 14. In this case, owing to the imposed constant hydrogen concentration at the surface, which is physically motivated^11^,^18^,^47^,^48^, the variation of surface energy with composition^44^ is neglected. However, the variation of surface stress with composition is included.

In this model, the average concentration is PdH_0.046_ while the net surface stress is tensile owing to a hydrogen-rich surface layer represented as a surface term and thus of zero width. A high hydrogen concentration surface layer is reasonable as enhanced hydrogen binding of the Pd surface and subsurface compared with the bulk was previously observed experimentally and in simulations^7^,^14^,^49^,^50^. A surface term can be used because the layer thickness is estimated to be on the order of 1 nm (ref. 14), which is below the spatial resolution of the experiment. The strain induced by this layer, however, extends throughout the particle. Figure 3b shows this causes expansion at the cube corners and, via stress-driven diffusion, relative hydrogen excess as well (Fig. 3c). This model matches well with the observed strain distribution. The measured strain distribution in pure Pd nanocubes (for example, with no prior hydrogen exposure) agrees with simulation results for nanocubes without any hydrogen (for a comparison see Supplementary Fig. 8). The experimentally measured α phase strain distributions are all different (Supplementary Fig. 9), which is consistent with differing distributions and concentrations of residual hydrogen and with the observation of residual strain after dehydriding in thin film strain studies^50^.

The hydrogen-rich surface layer in the α phase dominates the nanocube strain distribution and could be due to sluggish H_2_ desorption kinetics at room temperature and pressure^51^ as bulk hydrogen diffusion in this system is exceptionally fast (<1 ms for a 100 nm particle)^50^. A slow room temperature process is corroborated by the strain distribution in a particle that was held at 50 °C for 2 h (Supplementary Fig. 10). Although the Pd particle in Supplementary Fig. 10 was previously hydrogen cycled, its strains are consistent with an absence of hydrogen, implying loss of the hydrogen layer given enough time at elevated temperature. This is an important observation for a practical room temperature hydrogen storage system. Finally, we studied the strain field evolution during the α to β phase transformation.

### Dynamics during the hydriding phase transformation

**Figure 4:**
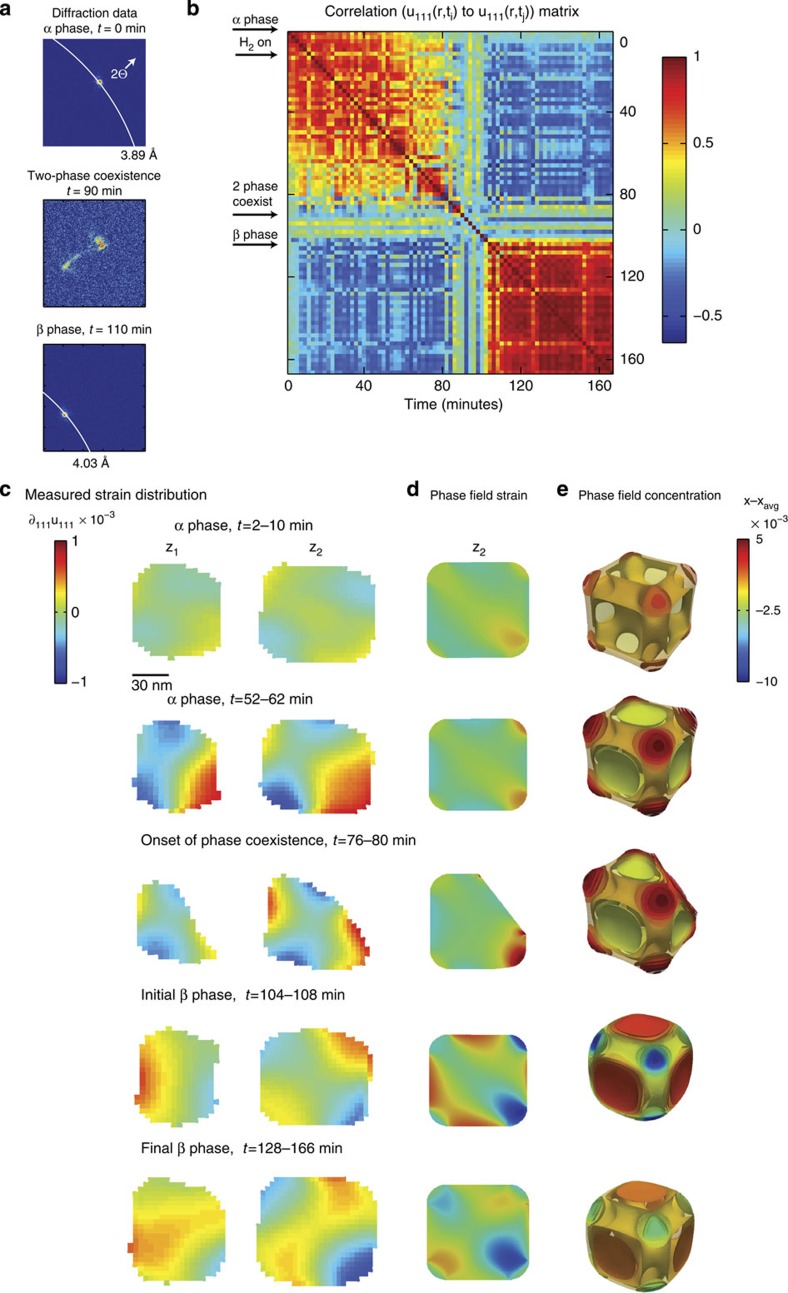
Strain evolution during the α to β phase transformation. (**a**) Diffraction data slices that show the α and β phase (111) Bragg peak positions, as well as an intermediate state showing phase coexistence. White arcs represent rings of fixed scattering angle. (**b**) The correlation matrix in which r_mn_ is the Pearson *r* correlation coefficient between *u*_111_(**r**,*t*=*m*) and *u*_111_(**r**,*t*=*n*). Arrows on the left hand side indicate the nanoparticle state. (**c**) The temporal evolution of ∂_111_*u*_111_ during the hydriding phase transformation at two cross-sections (for their spatial location, see Supplementary Fig. 6). (**d**) The phase-field strain distribution. (**e**) The concentration distribution deformed by the relative displacement field magnified by a factor of 500.

Figure 4a shows the location of the (111) diffraction peak on the detector. The experimental geometry is such that a ring of fixed scattering angle traces an arc from the upper left to the lower right of the detector, as shown by a white arc. At *t*=0, the α phase is characterized by a single peak at a 2*θ* angle corresponding to a lattice constant of 3.89 Å. At *t*=10 min, a gas mixture containing hydrogen is turned on and the Pd nanocube uptakes hydrogen. At *t*=90 min, two Bragg peaks are seen. The peak at approximately the same angle as at *t*=0 corresponds to the α phase, while the second peak at smaller 2*θ* (corresponding to a larger lattice constant) corresponds to a lattice-expanded phase. The fact that both peaks appear simultaneously on the detector indicates that two phases are simultaneously present in the single nanocube. We measured two-phase coexistence in single-particle diffraction data for six additional particles (for an additional example, see Supplementary Fig. 11). Intensity at intermediate 2*θ* values between the two peaks corresponds to crystalline regions of the crystal that have a lattice constant between the α and β phase. Only the β phase peak remains when the phase transformation is complete.

To quantify the changes in *u*_111_(**r**,*t*), we computed the Pearson *r* correlation coefficient


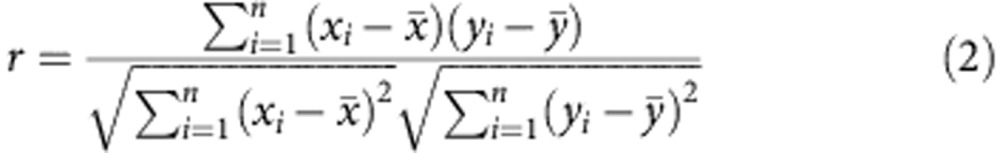


where *x*_*i*_ are the displacement field values for a particular pixel at time t, *y*_*i*_ are the displacement field values at the same spatial location at a different time t′, 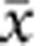 is the mean value of the displacement field at time t, and 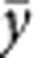 is the mean value of the displacement field at time t′. The sum is evaluated over the 3D displacement field and *r* is computed for all possible combinations of displacement fields. Figure 4b shows the correlation matrix, r_mn_, in which each entry is the correlation coefficient between *u*_111_(**r**,*t*=*m*) and *u*_111_(**r**,*t*=*n*). The matrix is symmetric and by definition has a value of unity along the diagonal.

Interestingly, the correlation time between α phase displacement field maps is a function of absolute time. For example, the first state is well-correlated to the subsequent 10 states while the twenty-fifth state is well-correlated only to the subsequent 5 states. This decay in correlation as a function of absolute time implies that the hydrogen adsorption dynamics are becoming faster, despite the constant hydrogen partial pressure, that the system shows aging^52^, and that hydrogen uptake may be described as autocatalytic. In addition to the slow decay in correlation, there are blocks of well-correlated measurements separated by uncorrelated periods. This signature is consistent with avalanches, or large intermittent changes during the dynamics^53^. Interestingly, the correlation time once the particle fully enters the β phase is relatively independent of absolute time.

Figure 4c shows the evolution of ∂_111_*u*_111_, computed from the average of the reconstructions corresponding to the five sets of highlighted points in Fig. 1d, as a function of time. Two cross-sections are shown for simplicity (for additional cross-sections see Supplementary Fig. 12). The strain distribution at *t*=2–10 min was previously shown and discussed (Fig. 3). At *t*=52–62 min, the strain map has changed in magnitude, although not in distribution. A higher average hydrogen concentration of PdH_0.12_ in the phase-field model explains the increase in strain magnitude.

The particle appears to undergo morphological changes, marked by the disappearance of Bragg electron density, during the onset of two-phase coexistence around *t*=76–80 min. The missing Bragg electron density in the reconstructed strain fields can occur in several ways. If a portion of the crystal rotates out of the Bragg condition, becomes amorphous, or undergoes restructuring to a different unit cell symmetry, this appears as missing Bragg electron density^31^,^35^,^54^. The most plausible explanation is that the β phase region nucleates at the corner of the cube and propagates inward, which is consistent with the location of the β phase region observed in the phase-field model (Supplementary Fig. 13). Interestingly, this demonstrates that phase propagation is preferred over phase nucleation as there is only one region of new phase observed that subsequently grows.

At *t*=104–108 min, the particle has gone through a phase transition and may be considered to be in the β phase but with a slight H deficiency. The average particle composition is now close to the β phase, and as such the net surface effect is compressive owing to positive surface energy. In this case, H is driven away from the corners towards the faces, and the same coupling of compositional inhomogeneity and strain occurs.

## Discussion

We have studied *in situ* 3D strain dynamics in single Pd nanocubes during the hydriding phase transformation using CXDI. We have used a full 3D phase-field model to aid in interpreting the experimentally measured strain distributions. The dilute hydrogen α phase strains are found to be particle dependent, consistent with trapped residual hydrogen and a tensile surface stress due to hydrogen adsorption. In the hydrogen-rich β phase, the strains are particle independent and consistent with elastic effects that tend to minimize surface area. During the phase transformation, structural two-phase coexistence is directly observed in the diffraction data. The structural correlations indicate that the phase transformation shows both ‘aging' and ‘avalanching'. The observed strain fields are consistent with hydrogen enrichment in the cube corners followed by the nucleation of a hydrogen-rich region at a single cube corner. Hydrogen excess in the α phase and deficiency in the β phase are seen to enhance the magnitude of the strain field via stress-driven diffusion. More generally, our results offer a new avenue to study phase transformations in single nanocrystals in reactive environments under operating conditions as a function of size, crystallinity and morphology.

## Methods

### Palladium nanocrystal synthesis

Palladium nanocubes were prepared according to Niu *et al.*^32^, with modifications. To make the initial Pd seed crystals, A 10 mM H_2_PdCl_4_ solution was prepared by dissolving 89 mg of PdCl_2_ (Aldrich, ≥99.9%) in 5 ml of 0.2 M HCl solution (Ajax Chemicals, AR grade) and further diluting to 100 ml with water (MilliQ, 18.2 MΩ cm^−1^). A measure of 1 ml of 10 mM H_2_PdCl_4_ solution was added to 20 ml of 12.5 mM cetyltrimethylammonium bromide (Unilab, 98%) solution heated at 95 °C under stirring (700 r.p.m.) in a 20 ml round bottom flask. After 5 min, 160 μl of freshly prepared 100 mM L-ascorbic acid (BDH Chemicals, 98.7%) solution was added. Twenty minutes after the ascorbic acid solution was added, a 160 μl aliquot of this as-synthesized nanocube seed solution and 500 μl portion of 10 mM H_2_PdCl_4_ solution and were added to 20 ml of 100 mM cetyltrimethylammonium bromide in a separate 50 ml round bottom flask. Freshly prepared 100 mM ascorbic acid solution (200 μl) was added following this, and the solution was mixed thoroughly. The resulting solution was placed in a water bath at 60 °C for 1 h. Then, a further 500 μl of 10 mM H_2_PdCl_4_ solution was added, followed by 200 μl of freshly prepared 100 mM ascorbic acid solution and the solution was well mixed. The flask was returned to the water bath at 60 °C and the reaction was stopped 1 h later by centrifugation (6,000 r.p.m., 10 min). Two more centrifugations (6,000 r.p.m., 10 min) were applied to the samples for transmission electron microscope (TEM) characterization and CXDI experiment sample preparation.

### TEM characterization

TEM images were acquired on a FEI Tecnai TF20 microscope operating at 200 kV. TEM samples were prepared by drop casting 20 μl of the Pd nanocube solution onto a copper TEM grid (300 carbon mesh) and drying in ambient conditions.

### Experiment sample preparation

CXDI samples were prepared by spin casting 200 μl of the Pd nanocube solution onto 15 × 15 mm silicon substrate at 2,000 r.p.m. for 60 s. This was then kept at 100 °C for 2 h.

### Coherent diffraction experiment details

A double crystal monochromator was used to select *E*=8.919 keV X-rays with 1 eV bandwidth and longitudinal coherence length of 0.7 μm. A set of Kirkpatrick Baez mirrors was used to focus the beam to ∼1.5 × 1.5 μm^2^. The rocking curve around the (111) Bragg reflection was collected by recording two-dimensional coherent diffraction patterns with a charge-coupled device camera (Medipix 3, 55 μm pixel size) placed at 0.26 m away from the sample around an angle of 2*θ*=36° (Δ*θ*=±0.5°). 2–3 full 3D data sets were taken for each palladium nanocrystal in a helium environment. The 3.6% mole fraction H_2_ gas in He was then mixed with the pure He gas to increase the partial pressure of H_2_ above zero. The pressure was left constant while 3D data sets were continuously collected at ∼2 min intervals for approximately 2.5 hours.

### Phase retrieval

The phase-retrieval code is adapted from published work^28^,^55^. The measured data with a random phase is used as the starting point for five independent reconstructions. Each reconstruction uses 90 iterations of the difference map and 10 iterations of error reduction repeated until 2,200 total iterations are reached^24^,^56^. The best reconstruction is chosen according to the lowest error metric. This reconstruction is used as the seed for the next generation in which five more independent reconstructions are run with the previously mentioned parameters. Five total generations, each with five members, are used^57^. The final resolution of 16 nm was computed via the phase-retrieval transfer function (Supplementary Fig. 4).

### Phase-field modelling

We model cubic PdH_*x*_ particles using coupled Cahn–Hilliard and elastic equations as established in Welland *et al.*^41^ The thermodynamic model assumes that PdH_*x*_ maintains the host Pd lattice structure while interstitial hydrogen is diffusing at an atomic fraction *x*. In the region of two-phase coexistence, *x* varies between equilibrium concentrations in the α and β phases, *x*^α^=0.017 and *x*^β^=0.60, respectively^1^. The local ratio of the β phase is modelled according to 
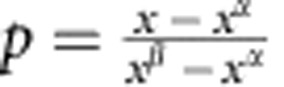
, which varies between 0 and 1 corresponding to the α and β phases, respectively. The system's free energy is described by


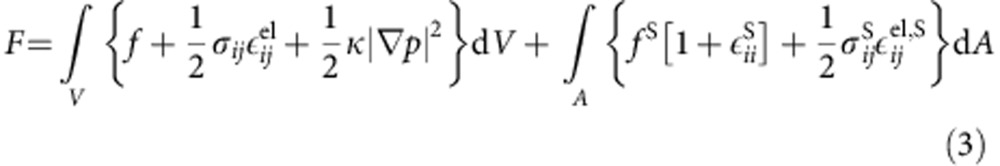


where *f* is the free-energy density containing both the enthalpy and the entropy, 
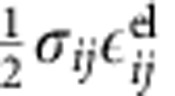
 is the elastic energy density, *p* is the local fraction of the β phase, and 
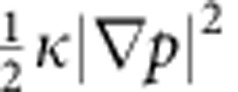
 is the gradient energy density. The surface contribution to the free-energy functional includes the surface energy contribution (in the Lagrangian system), which is a product of undilated surface energy and surface dilation term, and an additional stress contribution accounting for the change in surface energy with strain.

The first approximation to the free-energy density is as a double well potential, *f*=*ρΩRT p*^2^(1−*p*)^2^ where *ρ*, *Ω*, *R* and *T* represent the concentration of Pd, potential barrier height, ideal gas constant and temperature, respectively. This approximation for the free energy is reasonable as long as *p* is close to 0 or 1. The parameter *Ω* is fit to the measured hydrogen partial pressure above PdH_*x*_ using the relation between volumetric chemical potential of interstitial hydrogen and the partial pressure of H_2_ vapour by 
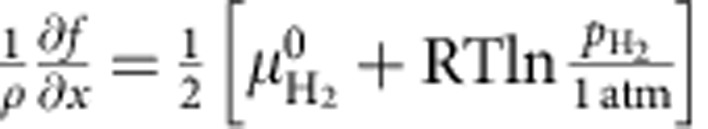
 (ref. 58). Using the free-energy potential above, 
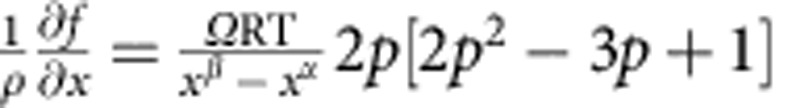
, is derived. Then, *Ω* is fit to the data of (ref. 59) above the β phase to 2.07 as shown in Supplementary Fig. 14.

The gradient energy coefficient *κ* is calculated from the potential barrier height and the desired interface thickness, *l*, as *κ*=2^−4^ *l*^2^ *Ω* (ref. 38). The interface thickness is chosen to be 10 nm for computational considerations. Auxiliary simulations were performed for PdH_0.046_ and PdH_0.60_ with smaller values and did not change the results. The total strain is obtained from the small deformation approximation of the displacement field *u*_*i*_ as 
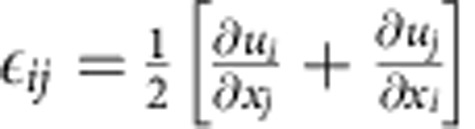
. The local elastic strain is 
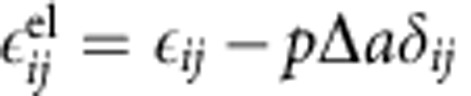
 according to Vegard's law where Δ*a* is the change in lattice constant between phases and *δ*_*ij*_ is the Kronecker delta. The elastic stress is given in the linear form, with the stiffness tensor linearly interpolated between phases, 
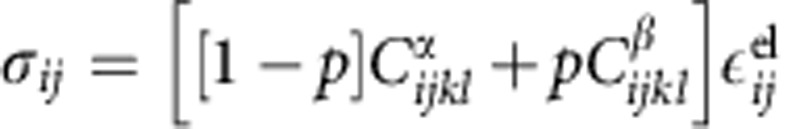
. The structural and elastic properties are summarized in Supplementary Table 1 (ref. 60).

Both surface terms implicitly include changes in hydrogen concentration near the surface. The model is derived under assumption that there is a surface tensile strain component with a maximum in the α phase, which decreases to zero in the β phase. This component is expressed through the strain difference with the β phase and can be ascribed to the effects of hydrogen enrichment of the surface layer with respect to the bulk concentration. This correlates well with both hydrogen monolayer adsorption, which was shown by previous calculations to be energetically favourable and with the models based on the hydrogen enriched layer that fit well with experimental observations. While hydrogen concentration is not explicit in determining the surface stress contribution, expansion of lattice parameter with increase in bulk hydrogen concentration is consistent with decrease of the tensile lattice stress. The surface energy *f*^S^ is taken to be that of (100) Pd, 0.866 J m^−2^ (ref. 61) and is scaled by the surface dilation 
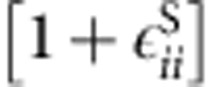
. Here, 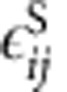 is the total surface strain given by 
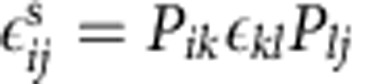
 and the surface projection operator is defined using the facet normal *n*_*i*_ as *P*_*ij*_=*δ*_*ij*_−*n*_*i*_*n*_*j*_.

It is well known that PdH_*x*_ surface can have higher H concentration than bulk. We have considered several models of an adsorbed hydrogen layer and describe here only the model that best matches experimental observations. In our model, we do not define the width of the layer (zero width). A physical interpretation of the model is either a surface hydrogen monolayer, which is found to be favourable in density functional theory calculations^49^ or a thin surface/subsurface hydrogen layer as observed in ref. 14. In both cases, the effect of the H layer on the bulk strain distribution is similar and is captured in the current model. The width of the layer is well below resolution of our X-ray experiments.

In the β phase, a surface layer of hydrogen is assumed to be at composition *p*=1. The surface elastic strain is then calculated as 
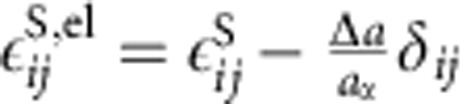
, and the surface stress 
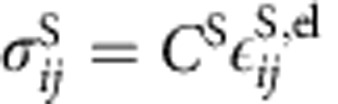
. The surface stress modulus, *C*^S^ is fit to produce the magnitude of the strain fields in PdH_0.046_. The fit value is 40 J m^−2^, which corresponds to a surface stress in the α phase of ∼1.3 J m^−2^, comparable to those listed in ref. 58. We define the surface stress as the derivative of the surface energy with respect to surface elastic strain (unitless). This follows the same form as ref. 58. In the β phase, the surface stress is zero and the observed strain field is attributed purely to surface area minimization from surface energy considerations.

### Phase-field governing equations

Assuming instantaneous elastic relaxation relative to diffusion, the displacement vector *u*_*i*_ is solved for quasi-statically within a virtual work formulation:


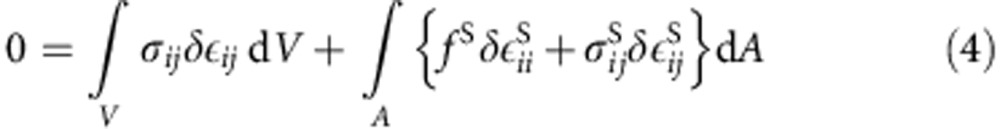


where the virtual strain 
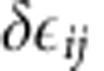
 has been projected onto the surface as described before. The chemical potential of interstitial hydrogen defined as


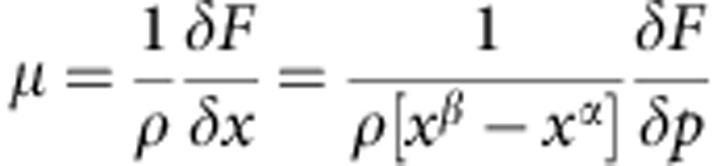


gives the governing equations for the volume and surface:


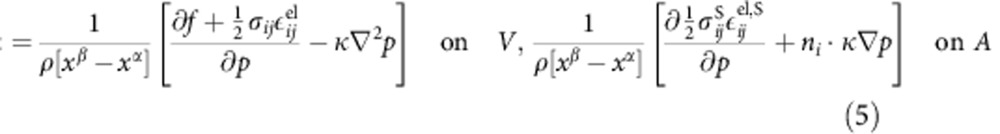


Currently, only the steady state, equilibrium situation is considered to describe strain distributions for different average *x*. However, the time-dependent case is solved for the sake of numerical robustness. The concentration of H evolves according to the Theory of Irreversible Processes as:


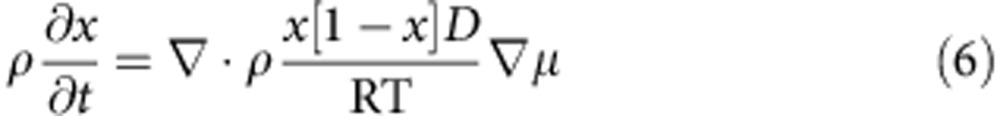


where the diffusion coefficient, *D*, is set to unity as it does not affect the equilibrium distribution. Equations 4, 5, 6 are solved for variables *u*_*i*_*, μ* and *x* using the finite element method implemented in the FEniCS package^62^,^63^,^64^,^65^. Simulations were run on a high-performance Linux cluster at Argonne National Laboratory using up to 256 cores.

## Additional information

**How to cite this article**: Ulvestad, A. *et al.* Avalanching strain dynamics during the hydriding phase transformation in individual palladium nanoparticles. *Nat. Commun.* 6:10092 doi: 10.1038/ncomms10092 (2015).

## Supplementary Material

Supplementary Information Supplementary Figures 1-14 and Supplementary Table 1
