# Supplementary material for: Avalanching strain dynamics during the hydriding phase transformation in individual palladium nanoparticles
Source: Nat Commun. 2015 Dec 11;6:10092. doi: 10.1038/ncomms10092 (PMC4682038; doi:10.1038/ncomms10092)
Supplement: Supplementary Information — Supplementary Figures 1-14 and Supplementary Table 1 [file ncomms10092-s1.pdf]

Supplementary Figures.

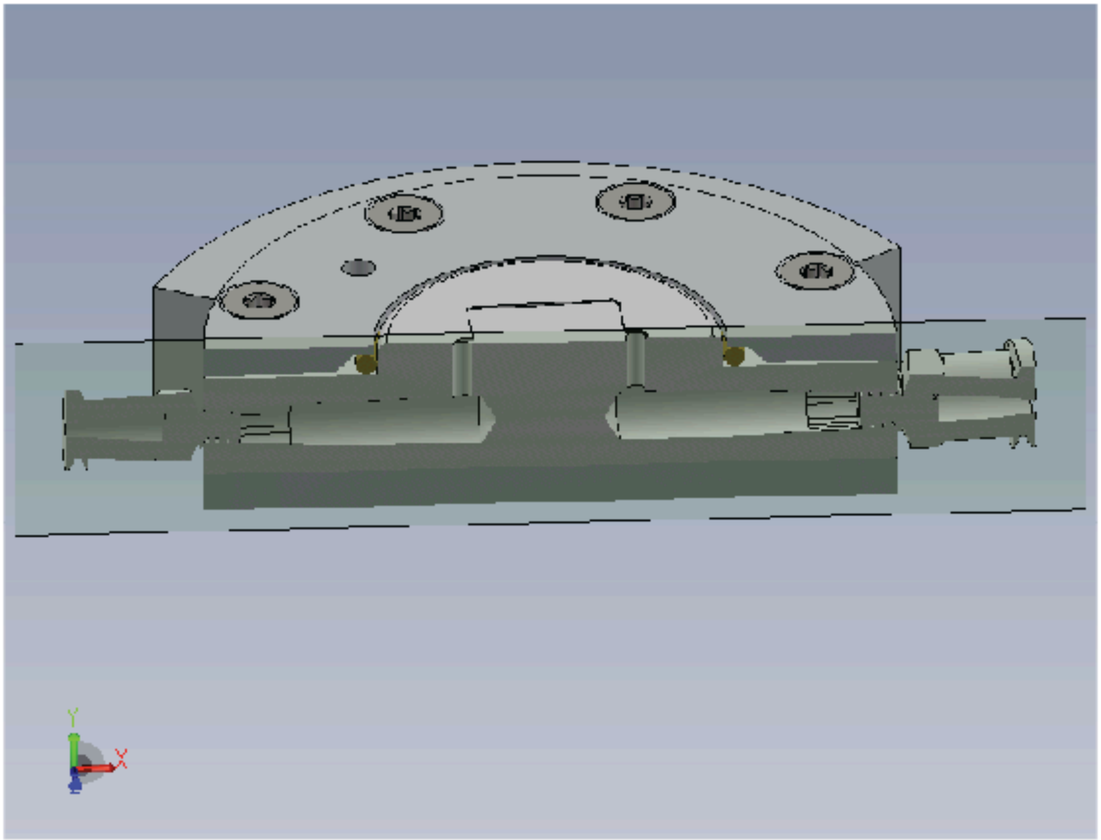

Supplementary Figure 1. Cutaway view of in-situ environmental gas cell. Gas flows into the side and up through channels onto the sample stage. A Mylar film allows x-rays to enter and exit the environment without significant reduction in intensity. Two valves (not shown) at the inlet and outlet are remotely actuated.

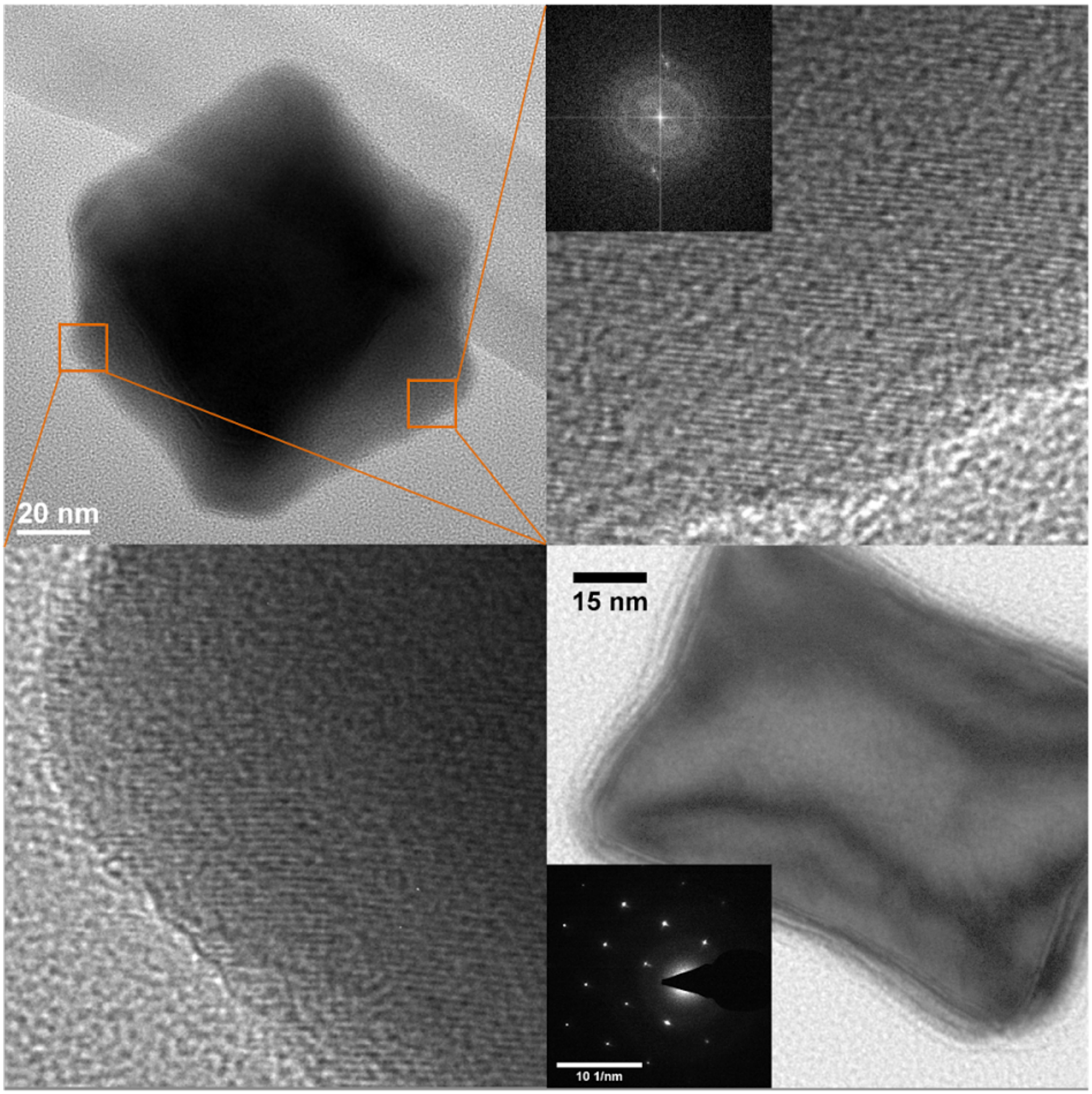

Supplementary Figure 2. Transmission electron micrographs of the sample. Palladium nanoparticles were dispersed on a copper TEM grid (300 carbon mesh). The top left part of the figure shows a cube at an angle that allowed lattice planes to be imaged, the bottom right is a different cube that is lying flat, i.e. with the {100} facet parallel to the plane of the grid. The inset in the top right image is the FFT of the area shown in the main image. Analysis of the data from this image yielded a lattice spacing of 3.9 Angstrom. The inset in the image below shows the single crystalline FCC electron diffraction pattern for the entire crystal shown in this image.

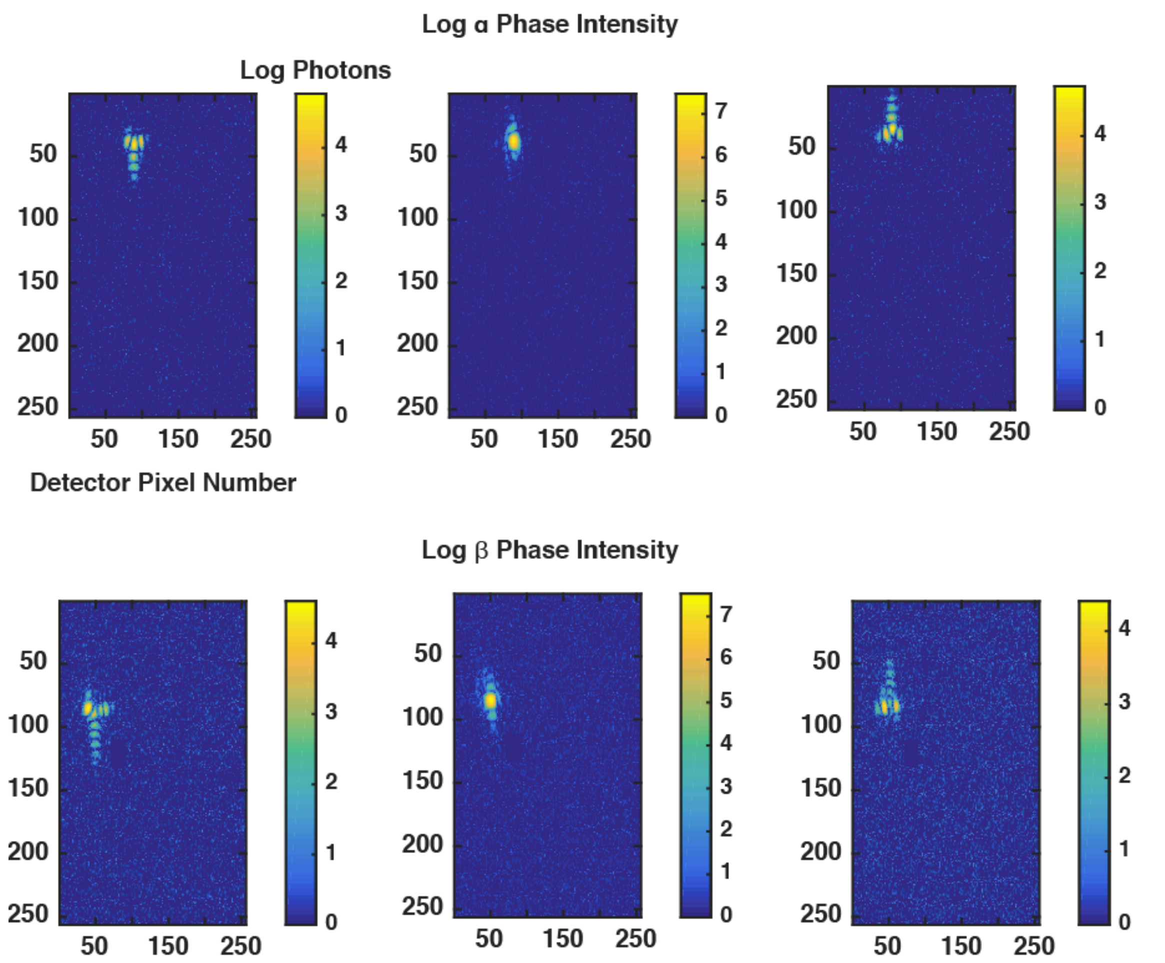

Supplementary Figure 3. Log of raw experimental data in both the α (upper row) and β (lower row) phases. Three different 2D slices corresponding to different sample rotations with respect to the incident x-ray beam are shown. The colorbars are the log of the number of photons. The reconstructions in the main text are averages of multiple data sets and thus have a higher signal to noise ratio than the single 3D measurements shown.

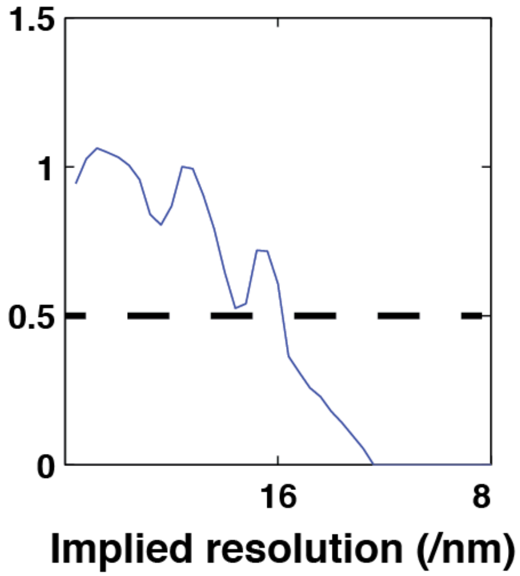

Supplementary Figure 4. Phase retrieval transfer function used to calculate the resolution of the reconstructed displacement fields. A cut-off of 0.5 was applied to determine the resolution of 16 nm.

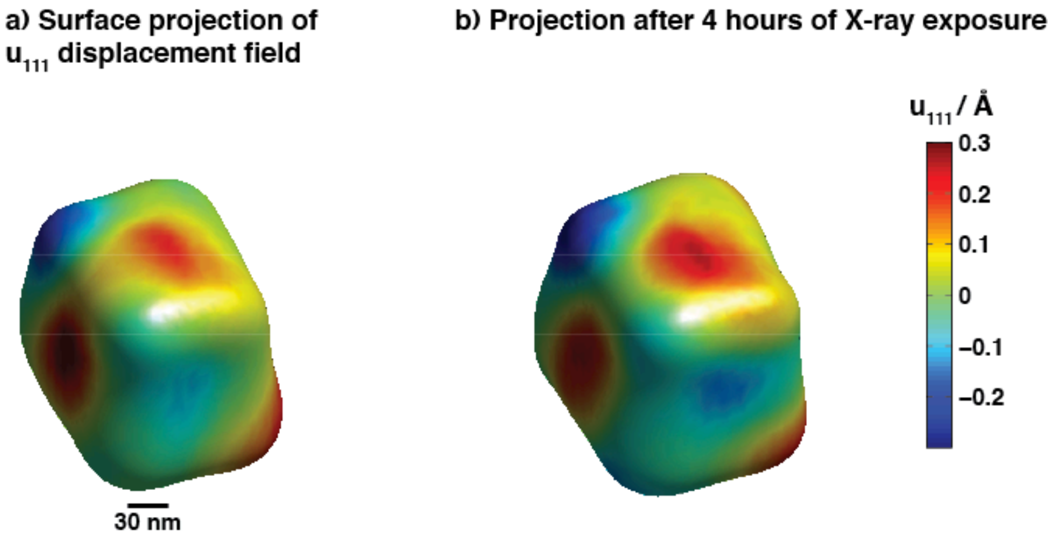

Supplementary Figure 5. Stability of Pd nanoparticles under x-ray exposure in helium environment. The u_{111} displacement field and particle shape is stable under x-ray exposure in a helium environment.

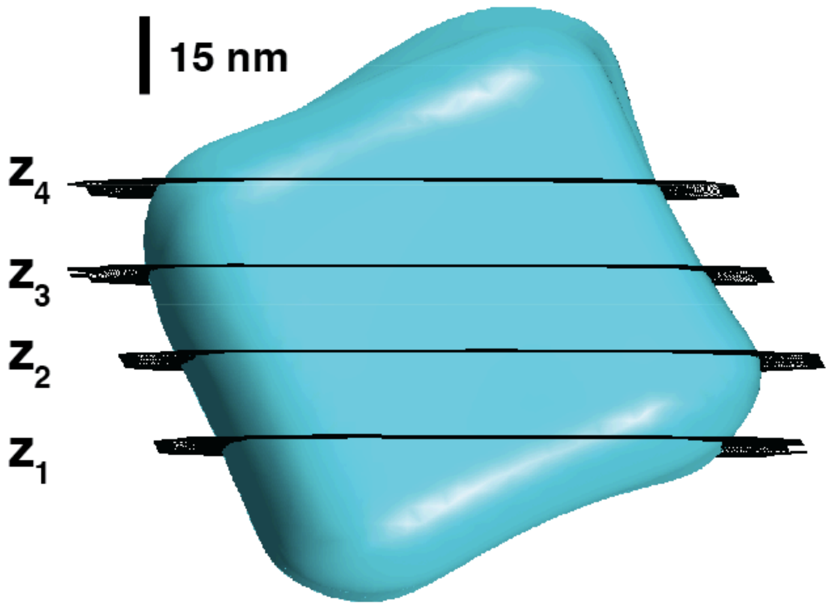

Supplementary Figure 6. Isosurface rendering of a particular nanocube. Black planes show the location of the 4 cross-sections discussed in Figures 2-3 and Fig. S6-7.

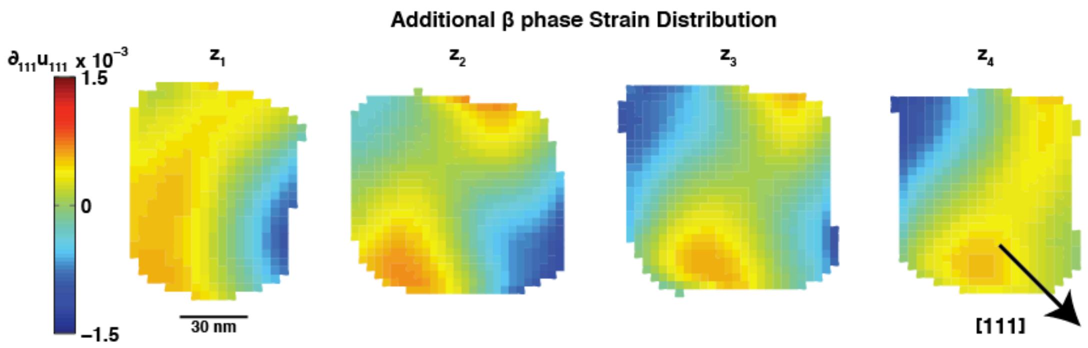

Supplementary Figure 7. Strain map in additional β phase particle. The strain distribution is consistent with purely elastic effects.

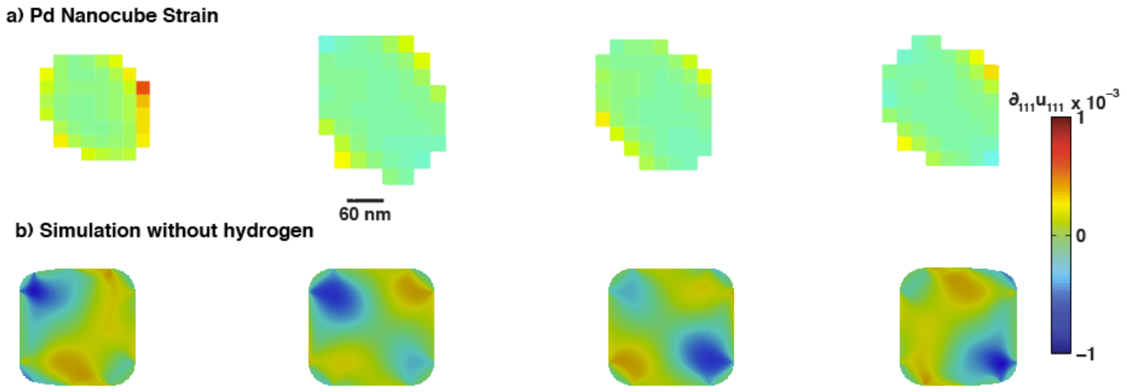

Supplementary Figure 8. Strain maps in a pure Pd nanocube compared to the phase field model without hydrogen. Both maps show compression at the body diagonal corners that lie along the scattering vector.

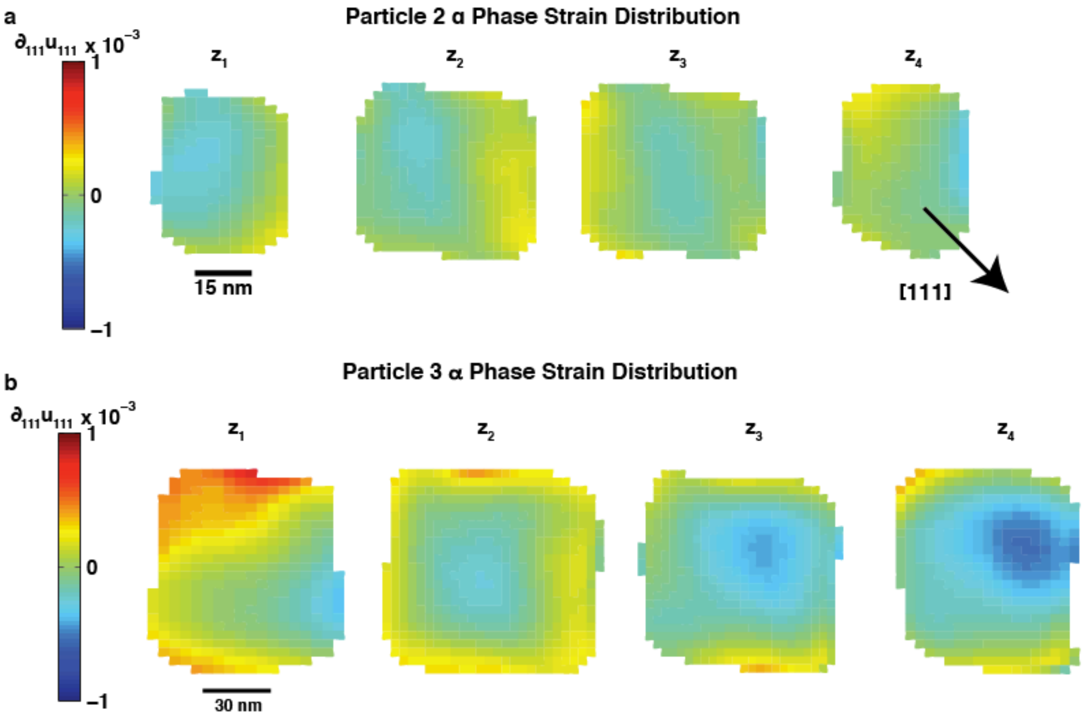

Supplementary Figure 9. Strain maps in additional alpha phase particles. The strain distributions are consistent with residual hydrogen. Both particles were previously hydrided and subsequently allowed to dry in air for 3 or more hours.

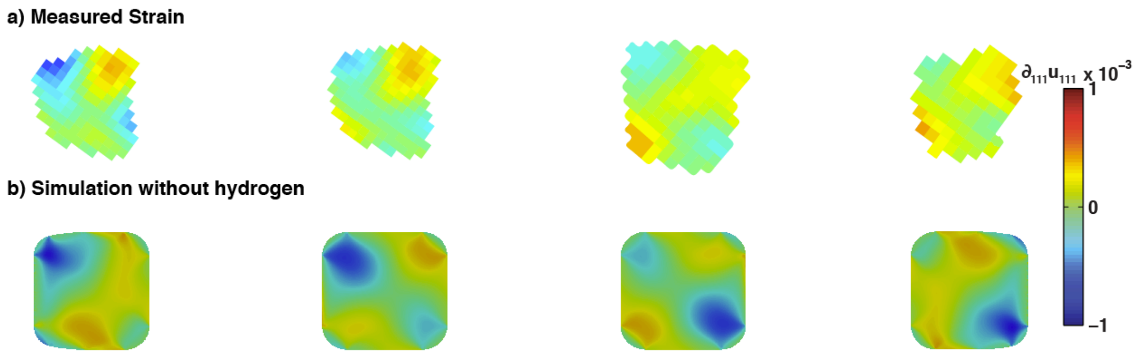

Supplementary Figure 10. Strain maps for an 85 nm α phase nanocube that was previously exposed to H and subsequently dehydrided in He at 50 °C for 2 hours. The experimental strain distribution resembles the calculated distribution in a pure Pd cube without hydrogen.

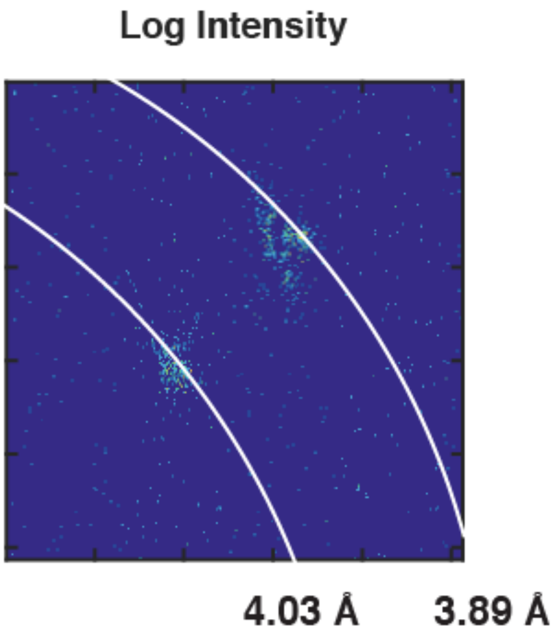

Supplementary Figure 11. Additional example of two-phase coexistence in single particle diffraction data. The Debye-Scherer rings corresponding to the α and β phases are drawn as white arcs. Although the data are not reconstructible, the simultaneous appearance of two peaks indicates two phases within a single particle.

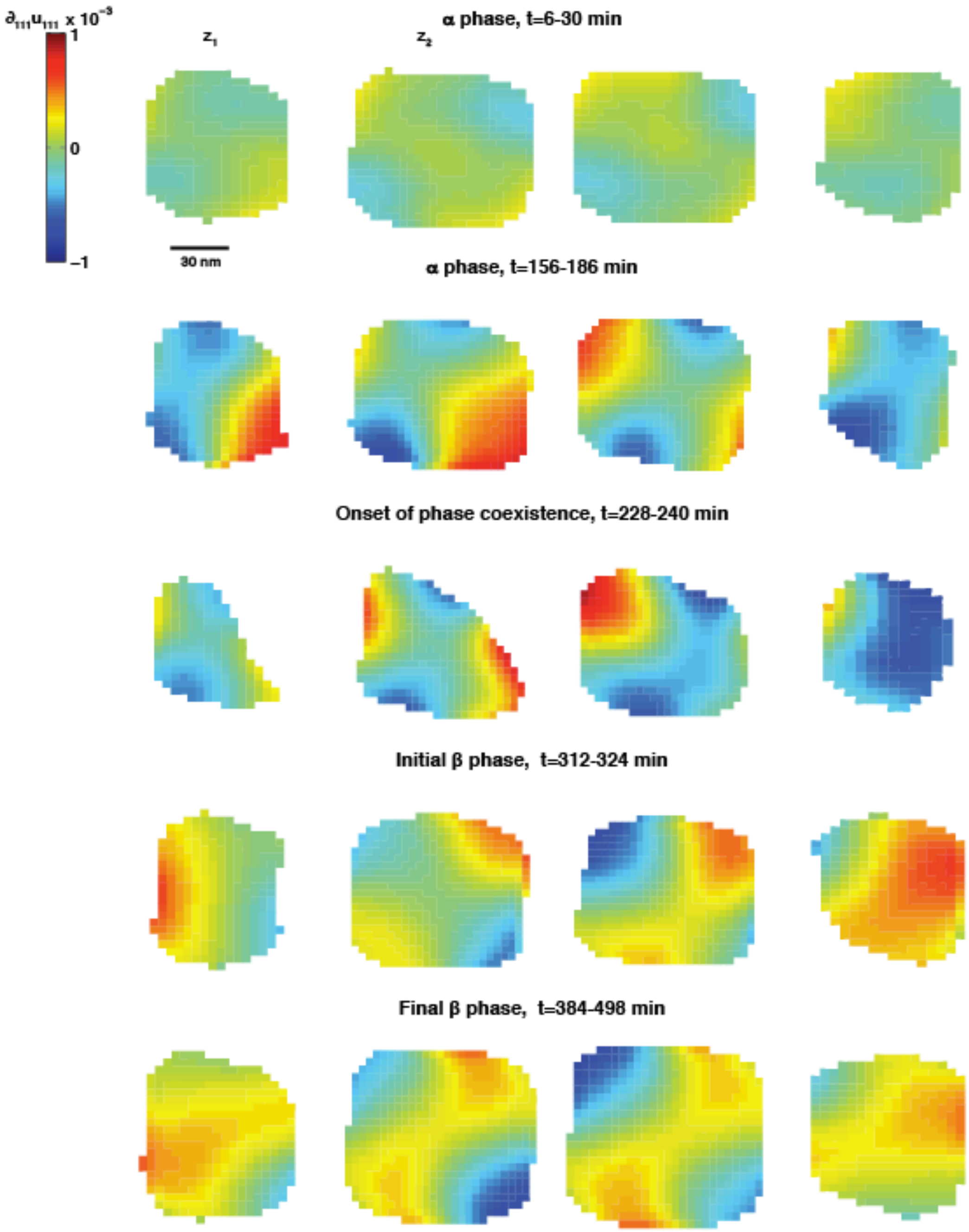

Supplementary Figure 12. Strain evolution during the phase transformation for 4 cross-sections. The cross-sections are taken at spatial locations as shown in Fig. S5.

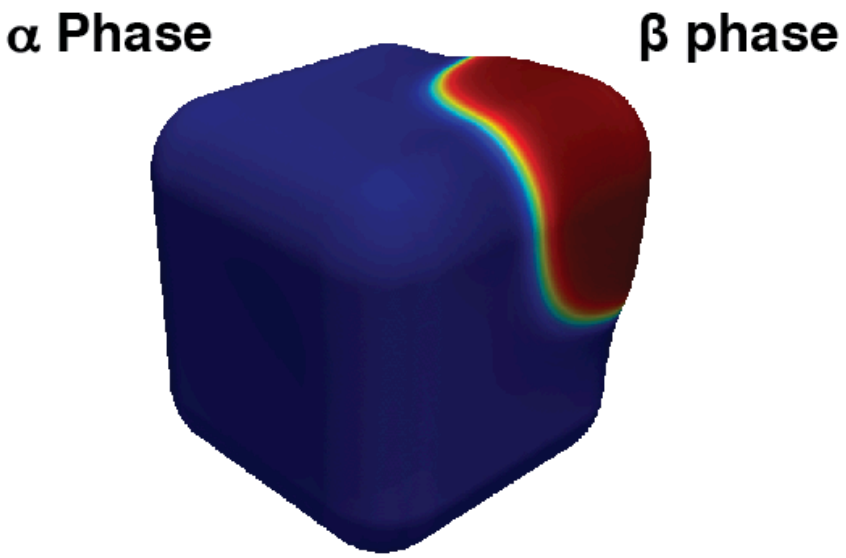

Supplementary Figure 13. Nucleation of the β phase during the phase transformation. The precipitate nucleates at the corner in the phase field simulation.

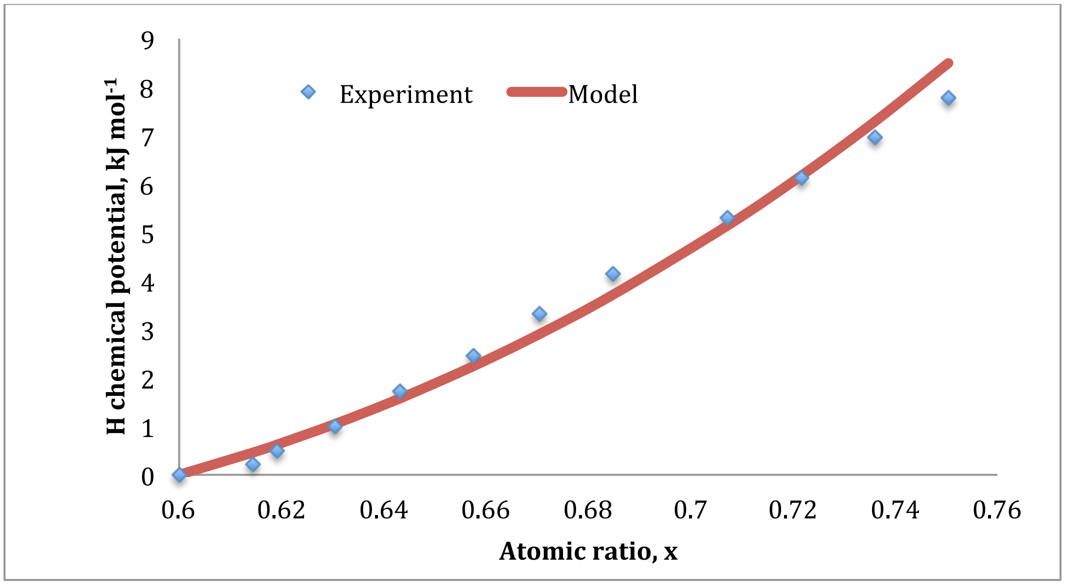

Supplementary Figure 14. Fit of the PdH chemical potential to experimental data. Chemical potential of H in PdH_x was fit to experimental data in [1].

	α	β	Source
a, Å	3.895	4.025	[1]
C_{11}, GPa	223	206	[64] from Pd and β-$PdH_{0.66}$ respectively.
C_{12}, GPa	173	152	
C_{44}, GPa	71	63	

Supplementary Table 1. Summary of structural and elastic properties used in the phase field model.
